# Analysis of the effect of platelet-derived growth factor combined with T cells in the diagnosis of hepatocellular carcinoma and prognostic assessment: A prospective cohort study

**DOI:** 10.5937/jomb0-60968

**Published:** 2026-04-15

**Authors:** Fei Wu, Guo Chen, Zuxin Wang, Youquan Niu, Wenqing Zhang, Hua Jiang

**Affiliations:** 1 The Third People's Hospital of Yunnan Province, Department of Occupational Diseases, Kunming, China; 2 Second Affiliated Hospital of Kunming Medical University, Cancer Treatment Centers, Kunming, China

**Keywords:** PDGF, Th17/Treg, liver cancer, D-TACE, combined detection, diagnosis, PDGF, Th17/Treg, rak jetre, D-TACE, kombinovana detekcija, dijagnoza

## Abstract

**Background:**

The aim of this study is to investigate the diagnostic and prognostic value of combined detection of serum platelet-derived growth factor (PDGF) and Treg/Th17 in hepatocellular carcinoma (HCC).

**Methods:**

We included 204 HCC patients managed with drug eluting beads-transcatheter arterial chemoembolization (D-TACE) and 100 healthy controls. Serum PDGF concentrations were measured via enzyme-linked immunosorbent assay (ELISA), while flow cytometry quantified Treg and Th17 cell proportions. The predictive performance of PDGF, Th17/Treg, and their combination for HCC diagnosis, therapeutic response, and 1-year overall survival (OS) was evaluated using receiver operating characteristic (ROC) analysis, Kaplan-Meier survival curves.

**Results:**

HCC cases exhibited notable elevations in serum PDGF concentrations and Th17/Treg than healthy controls, with tumor tissue PDGF expression significantly correlating with serum levels (P &lt; 0.05). In addition, we found a positive correlation between PDGF, Th17/Treg and tumor markers in HCC patients (P &lt; 0.05). PDGF + Th17/Treg detection yielded an area under the curve (AUC) of 0.835 (68.14% sensitivity, 82.00% specificity), surpassing individual markers. For predicting D-TACE non-response (progressive disease, PD), the combined detection showed 71.70% sensitivity and 84.11% specificity. The 1-year OS rates of the high PDGF group and the high Th17/Treg group were 75.19% (97/129) and 61.22% (30/49), respectively, while the 1-year OS rates of the low PDGF group and the low Th17/Treg group were 92.00% (69/75) and 87.74% (136/155), respectively (P &lt; 0.05). The AUC of the combined model for predicting death was 0.705.

**Conclusions:**

PDGF combined with Th17/Treg showed excellent diagnostic value for the occurrence and poor prognosis of HCC.

## Abstract (sr)

**Uvod:**

Cilj je ispitivanje dijagnostičke i prognostičke vrednosti kombinovane detekcij'e serumskog faktora rasta izvedenog iz trombocita (PDGF) i Treg/Th17 kod hepatocelularnog karcinoma (HCC).

**Metode:**

Uključili smo 204 pacjenta sa HCC lečenih transkateterskom arterjskom hemoembolizacjom sa lekom (D-TACE) i 100 zdravih kontrola. Koncentracij'e serumskog PDGF merene su imunosorbentnim testom povezanim sa enzimima (ELISA), dok j'e protočna citometrij'a kvantifiko-vala proporcije Treg i Th17 ćelija. Prediktivna efikasnost PDGF, Th17/Treg i nj'ihove kombinacij'e za dij'agnozu HCC, terapijski odgovor i jednogodišnje ukupno preživljavanje (OS) procenjena je korišćenjem ROC analize i Kaplan-Maj'erovih krivih preživlj'avanj'a.

**Rezultati:**

Slučajevi HCC-a pokazali su značajno povećanje koncentracij'a PDGF u serumu i Th17/Treg u poređenj'u sa zdravim kontrolama, pri čemu j'e ekspresij'a PDGF u tumor-skom tkivu značaj'no korelirala sa nivoima u serumu (P &lt; 0,05). Pored toga, pronašli smo pozitivnu korelacju između PDGF Th17/Treg i tumorskih markera kod pacjenata sa HCC-om (P &lt; 0,05). Detekcij'a PDGF + Th17/Treg dala j'e površinu ispod krive (AUC) od 0,835 (senzitivnost 68,14%, specifič-nost 82,00%), prevazilazeći pojedinačne markere. Za pred-viđanje neodgovora na D-TACE (progresivna bolest, PD), kombinovana detekcija je pokazala osetljivost od 71,70% i specifičnost od 84,11%.

**Zaključak:**

PDGF u kombinaciji sa Th17/Treg pokazao je odličnu dijagnostičku vrednost za pojavu i losu prognozu HCC-a.

## Introduction

Hepatocellular carcinoma (HCC) is a leading contributor to global cancer-associated deaths, with its incidence progressively increasing and outcomes remaining dismal [1]. How to achieve rapid and accurate diagnosis and prognosis evaluation of HCC is still a hot and difficult point in modern clinical research [2]. However, existing predictive models mostly rely on imaging evaluation (such as modified Response Evaluation Criteria in Solid Tumors [mRECIST]), traditional serum markers (e.g. alpha-fetoprotein [AFP]), or clinicopathological features, resulting in insufficient sensitivity and specificity.

In recent years, the role of tumor microenvironment (TME) in HCC carcinogenesis, progression, and therapeutic responses has garnered growing attention [3]. Platelet-derived growth factor (PDGF), a critical mediator of angiogenesis and fibrosis, is highly expressed in HCC [4]. Increased serum PDGF expression has been associated with enhanced invasion, vascular infiltration, and metastasis as well as poor prognosis in HCC, possibly by promoting tumor angiogenesis, enhancing cancer cell proliferative, invasive, and migrative potentials, and shaping the immunosuppressive microenvironment [5]. On the other hand, T cell-driven immunity is vital for HCC control, with T lymphocyte dynamics and functional status influencing disease progression and treatment efficacy [6]. However, current studies primarily focus on individual biomarkers [7][8], with limited exploration of how PDGF (pro-tumor) and T cells (anti-tumor) jointly predict HCC progression.

Despite the crucial role of both PDGF and T cells in HCC development, the feasibility of serum PDGF expression combined with peripheral blood T cell dynamics as a robust and independent predictor of therapeutic response and survival after D-TACE remains to be systematically studied. This study introduces and validates a novel biomarker combination—»serum PDGF + peripheral blood T cells«—for predicting post-D-TACE treatment response. Unlike single-dimensional approaches, this model integrates two critical biological processes (PDGF-driven tumor progression and T cell-mediated immunity), offering a more comprehensive prognostic assessment. Additionally, investigating their dynamic interplay and association with radiological/survival endpoints could enhance our understanding of post-D-TACE TME alterations.

## Materials and methods

### Research subjects

**Figure 1:**
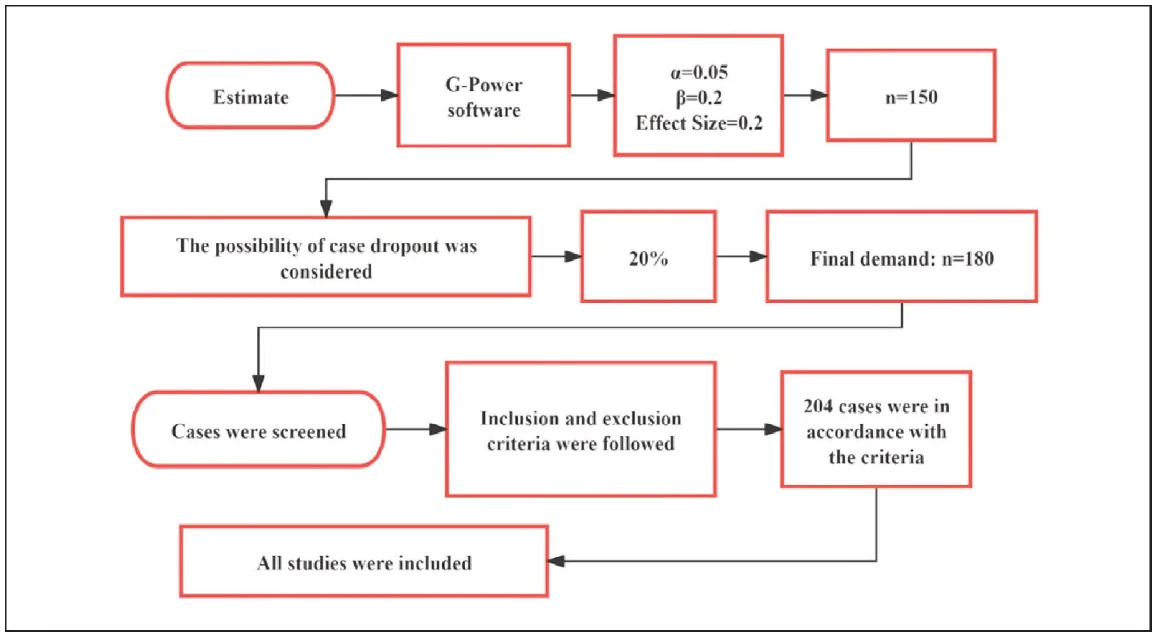
Sample Size Estimation Results. Sample size Determination Based on Power Analysis. Set α = 0.05, β = 0.2, and Effect Size = 0.2 based on prior PDGF studies in HCC [11], we calculated that the total sample size should be 180. In addition, the possibility of 10% shedding was also considered. A total of 204 HCC patients and 100 healthy controls were enrolled, which met the statistical requirements.

Based on sample size estimation results (Figure 1), 204 HCC patients were included as the research participants. Inclusion criteria: Participants aged 18-75 years with Child-Pugh A or B [9] were included; HCC diagnosis was established through imaging or histopathology; An ECOG performance score [10] of 0 or 1 was required, underwent radical resection of HCC in our hospital, and drug eluting beads-transcatheter arterial chemoembolization (D-TACE) was performed 4 weeks after surgery. Exclusion criteria: Individuals who underwent D-TACE or received immunomodulatory/targeted agents were excluded; Those with concurrent malignancies or autoimmune diseases were ineligible; Impaired hepatic/renal function (eGFR <60 mL/min) led to exclusion. In addition, 100 healthy controls matched by age and sex to the HCC patients were enrolled during the same period. Inclusion criteria for controls included an age range of 18-75 and no major medical history (based on health screenings at our center). Exclusion criteria for controls comprised liver dysfunction (ALT > 40 U/L or AST > 40 U/L) or other malignant diseases detected during health examinations. This study received ethical approval from our hospital's review board, and all participants provided written consent. Research procedures adhered to the Helsinki Declaration.

### Treatment plan and evaluation

All patients underwent radical resection and D-TACE in our hospital. The Seldinger technique was employed for femoral artery access in HCC patients, with subsequent superselective catheterization of tumor-feeding arteries [11]. Embolization was then performed using adriamycin-loaded microspheres (DEB, 100-300 mm) until blood flow cessation. Therapeutic outcomes were measured after 4-6 weeks per the mRECIST criteria [12], including complete response (CR), partial response (PR), stable disease (SD) and progressive disease (PD), disease control rate (DCR) =CR+PR+SD.

### Laboratory testing

Fasting venous blood (5 mL) was taken from two subject groups at admission and from HCC patients after D-TACE. After centrifugation (3000 rpm, 15 min), serum was collected, aliquoted into PE tubes, and frozen at -80°C. Subsequently, the ELISA technique was utilized to measure serum PDGF levels as per the kit instructions (Cusabio, CSB-E04687h). 100 μL of capture antibody working solution was added to each well and coated overnight at 4°C. The coating solution was discarded and 200 μL blocking solution was added to each well and blocked for 1h at 37°C. The standard was serially diluted from high concentration to low concentration according to the manufacturer's instructions, and 50 μL of standard/sample to be tested was added to each well and incubated at 37°C for 1h. The liquid in the Wells was discarded and washed 5 times with washing solution (PBST). Then 100 μL TMB substrate solution and 50 μL termination solution were added to each well, and the absorbance value (450 nm) was detected by microplate reader. Quality control: Internal controls (low/medium/high) with interassay CV<10%.

Additionally, cancerous and adjacent non-cancerous tissue samples (50 mg each) were obtained intraoperatively. After thorough lysis with 500 mL RIPA lysate, the samples were centrifuged (12000 rpm, 15 min) to obtain supernatants, followed by protein concentration determination using BCA and adjustment to 2 μg/mL. PDGF expression in tissues was detected according to the above scheme. For PBMC isolation, heparinized peripheral blood (5 mL) was diluted with PBS (5 mL), layered onto Ficoll separation medium, and centrifuged (400 Xg, 30 min). After Trypan blue staining, cells were resuspended (1x10^7^/mL). Tregs (CD4-FITC/CD25-PE/FoxP3-APC) and Th17 (CD4-APC/IL-17A-PE) were quantified by flow cytometry (BD FACS Canto II). CD4-FITC (Clone RPA-T4), CD25-PE (Clone M-A251), FoxP3-APC (Clone 236A/E7), IL-17A-PE (Clone eBio64DEC17). Gating: Live CD4^+^ FoxP3 + for Treg; IL-17A^+^ for Th17.

Blood samples of HCC patients at admission were used to detect tumor markers. The automatic immunoluminescence analyzer (Roche Cobas 8000) was used to preheat the instrument after startup and perform the instrument self-test. Load calibrators, quality control products, test antibodies, washing solution and substrate solution to the corresponding position of the instrument. The test samples, calibrators (3 concentration points: 0, 20, 100 mAU/mL), and quality controls (low value, high value) were loaded into the sample rack. The detection results of abnormal prothrombin (APT), AFP and α-L fucosidase (AFU) were obtained automatically. Quality control: Two horizontal quality control products were tested daily, and CV 8% (low value) and CV 10% (high value) were required.

### Prognosis follow-up

Patients were followed up for 1 year from the initial D-TACE, involving monthly check-ups, and one-year overall survival (OS) data were collected.

### Endpoints

Differences in peripheral blood PDGF expression and Th17/Treg between HCC patients and healthy controls were analyzed. In addition, the relationship between PDGF, Th17/Treg and tumor marker (AFP, APT, AFU) in HCC patients was observed. Additional analyses assessed the diagnostic potential of these biomarkers for HCC, as well as their ability to predict D-TACE response and OS outcomes.

### Statistical methods

All statistical analyses were performed using SPSS v25.0. Continuous variables underwent normality testing (Shapiro-Wilk) and were all expressed as (x̄±s), with group comparisons employing independent samples t-tests (inter-group) and paired t-tests (intra-group). Multiple comparisons (e.g., T0/T1/T2 biomarker levels) used Bonferroni correction (α = 0.0167 for 3 comparisons). Categorical data n(%) were analyzed via χ^2^ tests. Correlation (Pearson) and diagnostic accuracy (ROC) were performed. The joint model was constructed by logistic regression. Survival rates were calculated via the Kaplan-Meier method, with between-group survival differences assessed by log-rank tests. Statistical significance was defined as P < 0.05.

## Results

### Clinical baseline data comparison

The comparison of clinical baseline data like age, gender, family disease history, etc. revealed no significant difference between HCC patients and healthy controls (P > 0.05). Standardized mean differences (SMD) for these variables were below 0.1 (Table 1), confirming balanced baseline characteristics and establishing the validity of intergroup comparisons.

**Table 1:** Comparison of baseline data of the study subjects.

	Control (n = 100)	HCC patients (n = 204)	t or χ^2^	P	SMD
Age (years)	60.24±4.88	59.33±5.83	1.348	0.179	0.046
Gender			0.800	0.371	0.089
Male	64 (64.00%)	141 (69.12%)			
Female	36 (36.00%)	63 (30.88%)			
Family History of HSS			0.852	0.356	0.092
Yes	10 (10.00%)	28 (13.76%)			
No	90 (90.00%)	176 (86.27%)			
Smoking			0.927	0.336	0.096
Yes	55 (55.00%)	124 (60.78%)			
No	45 (45.00%)	80 (39.22%)			
Drinking alcohol			0.375	0.541	0.061
Yes	38 (38.00%)	85 (41.67%)			
No	62 (62.00%)	119 (58.33%)			

### PDGF and Th17/Treg in HCC

**Figure 2:**
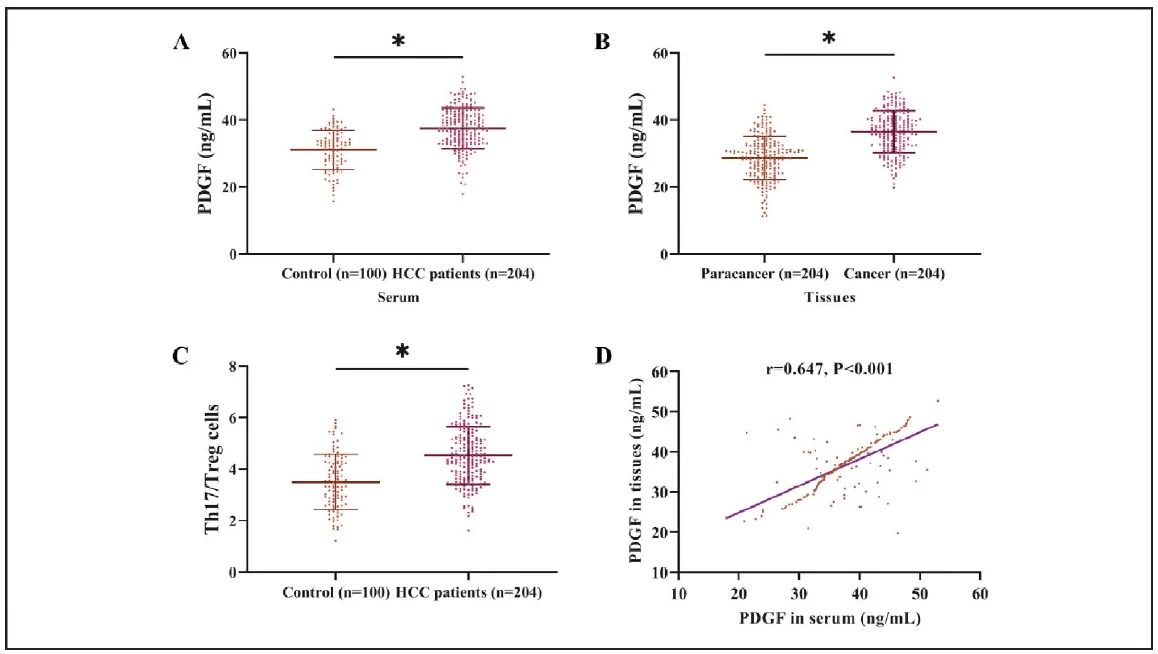
PDGF and Th17/Treg in HCC. (A) Comparison of serum PDGF concentrations. (B) PDGF expression in tissues. (C) Th17/Treg. (D) Correlation between serum PDGF and cancer tissue PDGF *P < 0.05.

Compared with healthy controls, serum PDGF increased significantly in HCC cases (P < 0.05, Figure 2A). Tissue analysis further revealed that PDGF expression was markedly upregulated in tumor tissues compared to adjacent non-cancerous tissues (P < 0.05, Figure 2B), suggesting the potential involvement of high PDGF expression in the occurrence and development of HCC. T-cell profiling demonstrated an elevated Th17/Treg in HCC cases versus healthy individuals (P < 0.05, Figure 2C), indicating disordered T cells in HCC. Correlation analysis showed that serum and tissue PDGF levels correlated strongly in HCC patients (P < 0.05, Figure 2D).

### Relationship between PDGF, Th17/Treg and tumor markers in HCC

**Figure 3:**
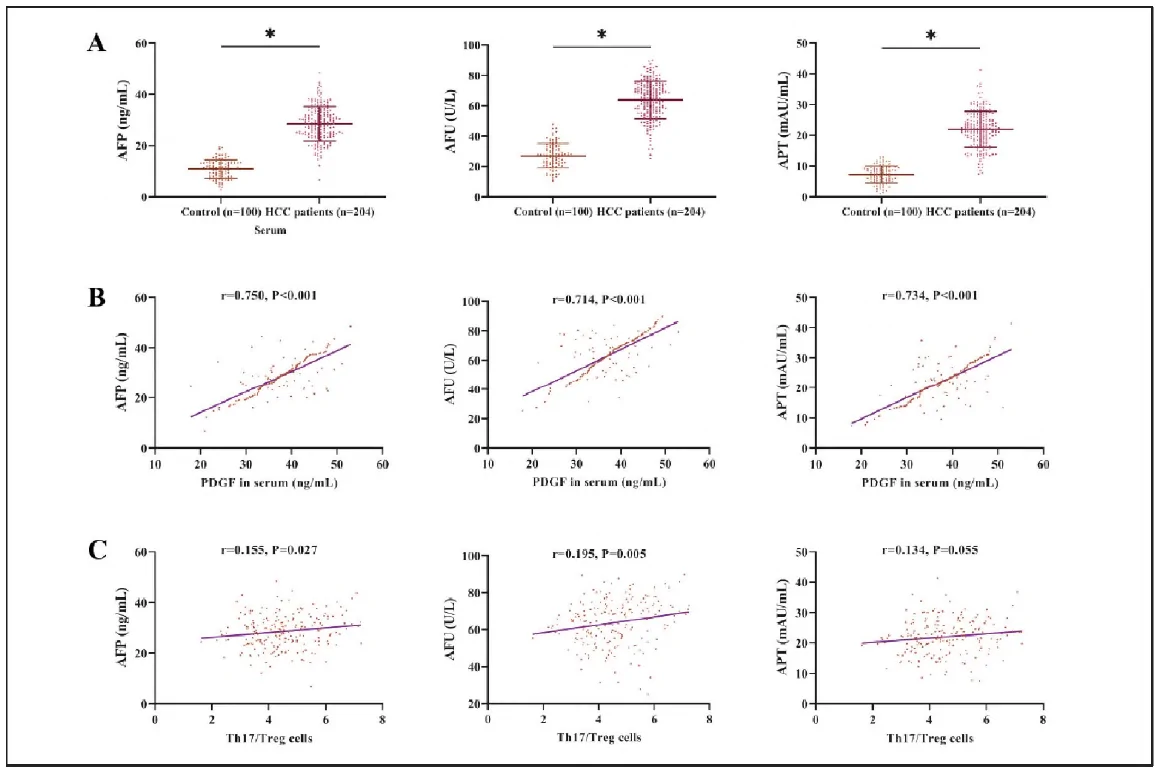
Relationship between PDGF Th17/Treg and tumor markers. (A) Comparison of tumor markers (AFP APT, AFU). (B) Correlation analysis of PDGF and tumor markers in HCC patients. (C) Correlation analysis of Th17/Treg and tumor markers in HCC patients. *P < 0.05.

AFF, APT and AFU were also significantly higher in HCC patients than in the control group (P < 0.05, Figure 3A). Subsequently, Pearson's correlation coefficient showed that PDGF and tumor markers (AFP APT, AFU) were all positively correlated in HCC patients (r=0.750, 0.714, 0.734, P < 0.05, Figure 3B). However, the correlation between Th17/Treg and AFU was not significant (r=0.155, 0.195, 0.134, P > 0.05, Figure 3C).

### Analysis of the diagnostic efficacy of PDGF and T cells in HCC

**Figure 4:**
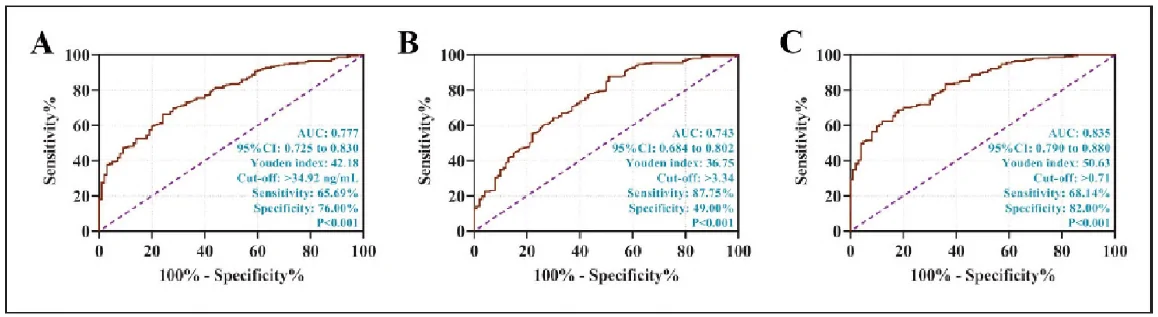
Diagnostic Efficacy Analysis. (A) ROC curve of PDGF alone in the diagnosis of HCC. (B) ROC curve of Th17/Treg diagnosis alone. (C) ROC curve of the combined model (PDGF+Th17/Treg).

According to ROC curve analysis, serum PDGF exhibited a diagnostic sensitivity of 65.69% and specificity of 76.00% for HCC (P < 0.05, Figure 4A), while Th17/Treg showed 87.75% sensitivity and 49.00% specificity (P < 0.05, Figure 4B). A combined diagnostic model integrating both markers was developed via binary logistic regression. The combined model [ Logit(P) = -8.686 + 0.173xPDGF + 0.877x Th17/Treg] significantly improved diagnostic power, achieving 68.14% sensitivity and 82.00% specificity (P < 0.05, Figure 4C). These findings highlight the model's strong diagnostic utility for HCC.

### Evaluation of the predictive efficacy of PDGF and T cells for treatment response in HCC

**Figure 5:**
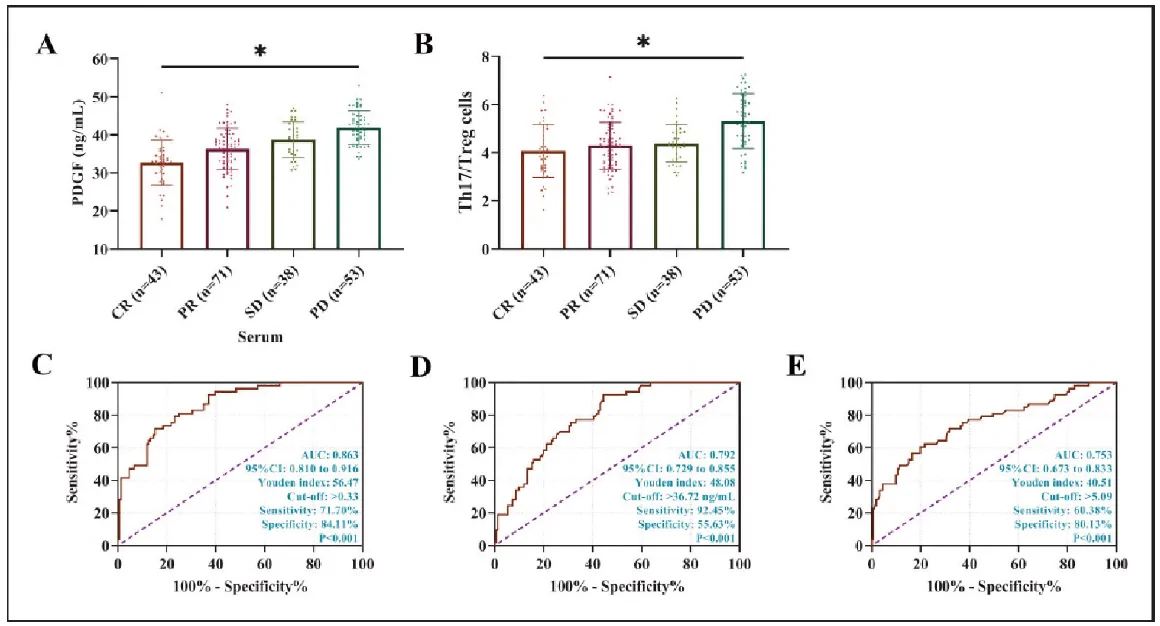
Prediction of Treatment Response. (A) Comparison of PDGF in patients with different therapeutic effects. (B) comparison of Th17/Treg in patients with different therapeutic effects. (C) ROC curve of the combined model for predicting PD. (D) ROC curve of PDGF for predicting PD. (E) ROC curve of Th17/Treg to predict PD. *P < 0.05.

Post-D-TACE evaluation showed a 74.02% DCR (151/204) in HCC patients. Responders (CR/PR/SD) exhibited significantly reduced PDGF expression and Th17/Treg versus non-responders (PD) (P < 0.05, Figure 5A, Figure 5B). The combined PDGF+Th17/Treg model [Logit(P)=-15.0.86+0.234xPDGF+ 1.025X Th17/Treg] achieved optimal PD prediction (71.70% sensitivity, 84.11% specificity; P < 0.05, Figure 5C), significantly surpassing individual biomarker performance (Figure 5D, E).

### Prognostic value of PDGF and Th17/Treg in HCC

**Figure 6:**
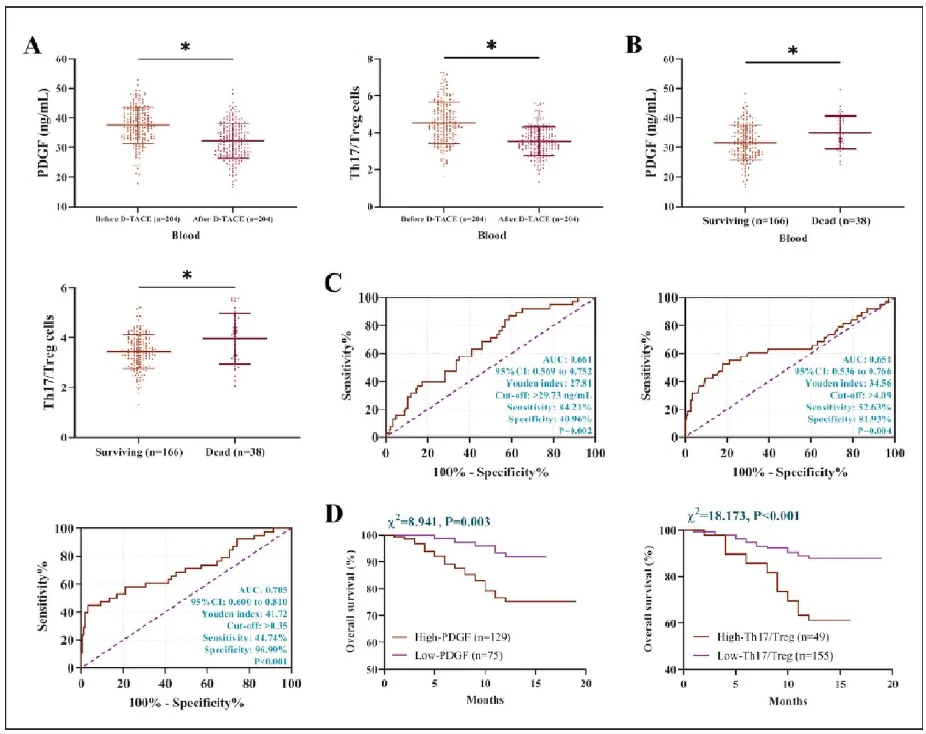
Prognostic Value Analysis. (A) Dynamic changes of PDGF and Th17/Treg before and after treatment. (B) biomarker levels in survivors versus nonsurvivors. (C) ROC curves of PDGF Th17/Treg, and combined models for predicting 1-year mortality. (D) Kaplan-Meier survival curves. *P < 0.05.

Post-treatment analysis revealed significant reductions in both PDGF levels and Th17/Treg compared to baseline measurements in HCC cases (P < 0.05, Figure 6A), further supporting their involvement in HCC progression. The median follow-up was [13][14] months, and the one-year OS rate was 81.37% (166/204). Notably, non-survivors exhibited significantly higher post-treatment PDGF and Th17/Treg levels than survivors (P < 0.05, Figure 6B). Post-treatment PDGF and Th17/Treg were used for prognostic prediction. The combined PDGF and Th17/Treg model [Logit(P) = -8.178 + 0.100xPDGF + 0.912x Th17/Treg] demonstrated excellent performance with 44.74% sensitivity and 96.99% specificity for one-year survival prediction (P < 0.05, Figure 6C). When patients were stratified by optimal cut-off values into high/low PDGF and Th17/Treg groups, Kaplan-Meier analysis revealed significantly worse survival outcomes in both high PDGF and high Th17/Treg groups compared to their low-expression counterparts (P < 0.05, Figure 6D).

## Discussion

Findings revealed markedly elevated PDGF levels and Th17/Treg in HCC patients, correlating strongly with disease progression, treatment resistance, and adverse survival outcomes. Their combination significantly enhanced diagnostic sensitivity and prognostic precision compared to single-marker analysis. These results support the potential of this biomarker combination for clinical application while highlighting the synergistic role of PDGF-driven TME modulation and Th17/Treg immune dysregulation in HCC pathogenesis.

As a key angiogenic factor, PDGF expression is strongly associated with tumor invasiveness [13]. This study demonstrated that serum PDGF concentrations in HCC patients were significantly elevated compared to healthy controls, closely mirroring PDGF expression in tumor tissues. This dual upregulation indicates multiple oncogenic mechanisms: (1) PDGF enhances endothelial cell migration and vascular permeability via VEGF/VEGFR2 signaling activation, facilitating tumor neovascularization [14]. (2) The PDGF-BB/PDGFRp axis activates hepatic stellate cells, triggering collagen deposition and extracellular matrix remodeling, which forms a physical barrier that hinders drug penetration [15]. (3) PDGF promotes Treg expansion through STAT3 pathway, and at the same time induces Th17 to secrete IL-17, which aggravates angiogenesis and forms a cancer-promoting microenvironment [16]. Notably, this study found decreased PDGF post-D-TACE and its close connection with patient prognosis, further confirming PDGF's critical role in tumor progression. On the other hand, the abnormal Th17/Treg elevation reflects the imbalance between pro- and anti-inflammatory immune responses in HCC patients. Mechanistically, Th17 cells drive TME remodeling by secreting IL-17A, which activates NF-B to upregulate angiogenic factors (e.g., IL-8, MMP-9) [17]. Treg cells suppress CD8+ T-cell activity via CTLA-4/LAG-3 overexpression, fostering local immune tolerance [18]. In this study, D-TACE induced a more pronounced Th17/Treg reduction in patients achieving CR, PR, or SD compared to PD cases. This suggests that serial Th17/Treg monitoring could track immunoediting dynamics and serve as a novel biomarker for assessing tumor immunogenicity. Moreover, the close relationship between PDGF, Th17/Treg and tumor markers also suggested the potential of these two markers as indicators for HCC disease assessment. Therefore, we aimed to confirm the diagnostic role of PDGF and Th17/Treg in HCC.

By integrating PDGF and Th17/Treg into a joint diagnostic model, this research achieved higher diagnostic precision for HCC. The synergistic effect emerges from their distinct mechanisms: PDGF captures tumor-specific traits, while Th17/Treg mirrors immune regulation. This approach outperforms single-marker methods in distinguishing HCC from cirrhosis and monitoring minimal residual disease (MRD). However, the lack of Child-Pugh grade-based subgroup analysis means the model's effectiveness across different liver function levels remains unverified, requiring additional studies. Meanwhile, their combination demonstrated significant prediction accuracy for D-TACE-nonresponsive patients, which may be due to the fact that biomarkers can reflect changes in tumor biological behavior earlier (at 4 weeks post-procedure). This early detection creates an opportunity for therapeutic modifications (such as sequential ablation therapy). Survival analysis showed that high PDGF and Th17/Treg cohorts exhibited 1-year OS rates of 75.19% and 61.22%, versus 92.00% and 87.74% in the low-expression group. The combined model was particularly effective in predicting prognostic death (AUC = 0.705), suggesting its potential as a biomarker for dynamic monitoring. Integrating the RECIST standard with the changing trend of serum markers is expected to realize »personalized management based on treatment response«.

Based on the above findings, we propose the following translational pathways: (1) PDGF combined with Th17/Treg can be used for high-risk screening in emergency department (cut-off>0.71, sensitivity 68.14%) or postoperative recurrence monitoring (monthly detection, cut-off>0.35). (2) Creation of a machine learning-driven dynamic monitoring system that synthesizes longitudinal biomarker trends and pre-/post-treatment radiomics to generate individualized prognostic models. (3) Investigation of combination therapies targeting PDGF inhibition + immune modulation, with prospective clinical trials focusing on patients exhibiting the high PDGF/Th17/Treg phenotype. Of course, due to the limited conditions, the limitations of this study can not be ignored. For example, the single-center retrospective design may lead to selection bias, warranting validation through multicenter cohorts. Although the sample size met the statistical requirements, it failed to cover all HCC molecular subtypes. Additionally, the absence of extended follow-up (>3 years) limits the assessment of long-term survival predictability.

## Conclusion

The combined detection of PDGF and Th17/Treg shows excellent diagnostic effect on the occurrence of HCC, and can accurately evaluate the prognosis of HCC patients after D-TACE treatment, suggesting that the combined detection of PDGF and Th17/Treg is a new and potential HCC disease evaluation scheme.

## Dodatak

### Availability of data and materials

The data that support the findings of this study are available from the corresponding author upon reasonable request.

### Acknowledgements

Not applicable.

### Conflict of interest statement

All the authors declare that they have no conflict of interest in this work.

### Contribution

Fei Wu, Guo Chen. These authors have the same contribution in this work.

## References

[1] Vogel A, Meyer T, Sapisochin G, Salem R, Saborowski A (2022). Hepatocellular carcinoma. Lancet.

[2] Morishita A, Tani J, Nomura T, Takuma K, Nakahara M, Oura K, Tadokoro T, Fujita K, Shi T, Yamana H, Matsui T, Takata T, Sanomura T, Nishiyama Y, Himoto T, Tomonari T (2021). Efficacy of Combined Therapy with Drug-Eluting Beads-Transcatheter Arterial Chemoembolization Followed by Conventional Transcatheter Arterial Chemoembolization for Unresectable Hepatocellular Carcinoma: A Multi-Center Study. Cancers (Basel).

[3] Wu M, Zhang Y, Sha B, Shu Q, Zhao L (2025). Preliminarna analiza dijagnostičke vrednosti i mehanizma dejstva TGFbI i S100A4 kod hepatocelularnog karcinoma. J Med Biochem.

[4] Wei C, Chen J, Yu Q, Qin Y, Huang T, Liu F, Pan X, Lin Q, Tang Z, Fang M (2024). Clonorchis sinensis infection contributes to hepatocellular carcinoma progression via enhancing angiogenesis. PLoS Negl Trop Dis.

[5] Moench R, Gasser M, Nawalaniec K, Grimmig T, Ajay A K, de Souza L C R, Cao M, Luo Y, Hoegger P, Ribas C M, Ribas-Filho J M, Malafaia O, Lissner R, Hsiao L (2022). Platelet-derived growth factor (PDGF) cross-signaling via non-corresponding receptors indicates bypassed signaling in colorectal cancer. Oncotarget.

[6] Huang C, Lao X, Wang X, Ren Y, Lu Y, Shi W, Wang Y, Wu C, Xu L, Chen M, Gao Q, Liu L, Wei Y, Kuang D (2024). Pericancerous cross-presentation to cytotoxic T lymphocytes impairs immunotherapeutic efficacy in hepatocellular carcinoma. Cancer Cell.

[7] Feng H, Li B, Li Z, Wei Q, Ren L (2021). PIVKA-II serves as a potential biomarker that complements AFP for the diagnosis of hepatocellular carcinoma. BMC Cancer.

[8] Cai L, Qiong Y, Chen S, Yan H (2022). Preoperativni odnos prealbumin-fibrinogen za predviđanje preživljavanja kod pacijenata sa hepatocelularnim karcinomom nakon hepatektomije. J Med Biochem.

[9] Tsoris A, Marlar C A (2025). Use Of The Child Pugh Score In Liver Disease.

[10] Shi Y, Han G, Zhou J, Shi X, Jia W, Cheng Y, Jin Y, Hua X, Wen T, Wu J, Gu S, Bai Y, Wang X, Zhang T, Chen Z, Zhang B, Huang M, Liu H, Mao Y, Zhou L (2025). Toripalimab plus bevacizumab versus sorafenib as first-line treatment for advanced hepatocellular carcinoma (HEPATORCH): A randomised, open-label, phase 3 trial. Lancet Gastroenterol Hepatol.

[11] Long F, Zhong W, Zhao F, Xu Y, Hu X, Jia G, Huang L, Yi K, Wang N, Si H, Wang J, Wang B, Rong Y, Yuan Y, Yuan C, Wang F (2024). DAB2+ macrophages support FAP+ fibroblasts in shaping tumor barrier and inducing poor clinical outcomes in liver cancer. Theranostics.

[12] Yu H, Bai Y, Xie X, Feng Y, Yang Y, Zhu Q (2022). RECIST 1.1 versus mRECIST for assessment of tumour response to molecular targeted therapies and disease outcomes in patients with hepatocellular carcinoma: A systematic review and meta-analysis. BMJ Open.

[13] Lu J, Hu Z, Yang M, Liu W, Pan G, Ding J, Liu J, Tang L, Hu B, Li H (2022). Downregulation of PDGF-D Inhibits Proliferation and Invasion in Breast Cancer MDA-MB-231 Cells. Clin Breast Cancer.

[14] Zou X, Tang X, Qu Z, Sun Z, Ji C, Li Y, Guo S (2022). Targeting the PDGF/PDGFR signaling pathway for cancer therapy: A review. Int J Biol Macromol.

[15] Wang J, Fang C, Noller K, Wei Z, Liu G, Shen K, Song K, Cao X, Wan M (2023). Bone-derived PDGF-BB drives brain vascular calcification in male mice. J Clin Invest.

[16] Sarri N, Wang K, Tsioumpekou M, Castillejo-López C, Lennartsson J, Heldin C, Papadopoulos N (2022). Deubiquitinating enzymes USP4 and USP17 finetune the trafficking of PDGFRβ and affect PDGF-BB-induced STAT3 signalling. Cell Mol Life Sci.

[17] Akhter S, Tasnim F M, Islam M N, Rauf A, Mitra S, Emran T B, Alhumaydhi F A, Ahmed K A, Aljohani A S M, al Abdulmonem W, Thiruvengadam M (2023). Role of Th17 and IL-17 Cytokines on Inflammatory and Auto-immune Diseases. Curr Pharm Des.

[18] Marangoni F, Zhakyp A, Corsini M, Geels S N, Carrizosa E, Thelen M, Mani V, Prüßmann J N, Warner R D, Ozga A J, di Pilato M, Othy S, Mempel T R (2021). Expansion of tumor-associated Treg cells upon disruption of a CTLA-4-dependent feedback loop. Cell.

